# On the Recurrence of Estruation in the Bitch after Oöphorectomy

**Published:** 1901-05

**Authors:** Cecil French

**Affiliations:** Washington, D. C.


### ON THE RECURRENCE OF ESTRUATION IN THE BITCH AFTER OÖPHORECTOMY.

By CECIL FRENCH, D.V.S.,
WASHINGTON, D. C.

AN impression prevails among certain of the laity that ablation of the ovaries is not invariably a preventative of the estrual function and the attendant uterine discharges. This idea is not altogether confined to the public at large, for it is shared by some members of the profession. We find Leeney,[1] a British practitioner, writing in 1890 to the effect that he could not understand how it was that spayed bitches and cats showed regular periods of estrum *unless operated upon when pregnant.* He instanced one case where the animal "took all the dogs in the parish" after the organs had been removed in their entirety, for he sent the ovaries and both cornua to the owner, a surgeon. In another instance a cat showed "heat" every three weeks after operation.

He had never seen maiden bitches exhibit any signs after operation. It is not quite clear what he meant by the term "maiden." Presumably he referred to bitches of any age which had never been allowed to mate, but it is possible he had in mind bitches which had not yet arrived at the age of puberty.

He proceeded to refer to a tradition existing among British farriers that a bitch should be allowed to copulate some ten days before being spayed, though none of them, on being asked, could offer any explanation.

Of later date Hobday makes the statement in his little handbook on *Canine and Feline Surgery* that the effect of the operation on estruation is by no means certain, and that he has on several occasions observed signs of estruation in bitches and cats whose ovaries had been *wholly* (the italics are mine) removed, the animals even copulating with the male.

In the past year Veterinarian E. J. Cary, of New York City, recently wrote me concerning a fox-terrier bitch whose ovaries he had removed in their entirety during the period between the first and second "heat." Soon after returning the animal to its owner, Cary was informed that it showed signs of "heat." Upon physical examination there were no signs of estruation, but when the animal was at large members of the opposite sex were continually torturing her by their persistent attentions, which were consistently repulsed. The condition continued for ten or twelve months when it ceased. No further report has been made to me.

None of the practitioners cited appears to have proceeded to investigate the matter. They made the observations and then let the matter rest. Cary endeavored to secure the privilege of conducting a second celiotomy, at my suggestion, but the owner declined to acquiesce.

Leisering,[2] writing in 1868, mentioned the case of an animal which "had been continuously very ruttish," after having been submitted to operation in earlier life. The necropsy showed that the operation had been bungled. The body of the uterus, left cornu, and left ovary had been removed, but a portion of the right horn, together with the right ovary, had been left behind. Both of the latter had undergone cystic dilatation, the ovarial tissue being degenerated.

In common with the practitioners cited I have also had similar experiences, which impelled me to follow up observation with investigation, so that some light might be thrown on this apparently remarkable phenomenon. Four instances of recurrent estruation after oöphorectomy have come to my immediate notice within the last few years. They were all in subjects upon which I had operated myself. Three of them were in dogs (fox-terriers) and the remaining one in a cat. The latter case[3] I have already referred to in the columns of this JOURNAL, but since then I have secured more minute details as to the animal's actions. I operated on this animal in March, 1899, removing the entire uterus, which contained six fœtuses, the oviducts, and, as I supposed, the ovaries in their entirety, after ligating the suspensory and other ligamentous tissue on their proximal extremities.

A few months later, about autumn, the subject exhibited a desire for sexual intercourse, which lasted some three weeks, reappeared early in the following spring, and then continued without cessation until last winter, when it became intermittent, and has appeared since about every three weeks. The desire is certainly decreasing in intensity. In my first account of this case I stated that the desire appeared a month after operation; that it had continued without intermission, and that the animal had never been seen in the act of coition. These were the particulars as I received them at that time from one member of the family, but which I find need modifying after more closely questioning the other members. It is now stated that this cat has been seen to copulate with various male cats on several different occasions, and that she becomes thinner in flesh as each "period" approaches. The owner refuses to submit her to a second operation for explorative purposes, but has promised me the privilege of conducting a necropsy in the event of her death. Consequently, I am unable to satisfy myself as to whether any ovarial tissue is remaining, but I fully expect to find such in the future.

Case No. 1 of the fox-terrier bitches was brought to me in June, 1896, for operation. The animal was born in the spring of 1895, and went through her first "period" about two months before operation. The operation in this instance was carefully performed, though only a cursory examination was made of the extirpated tissues. In the following October (1896) the owner informed me that the animal was again in heat and discharging. I advised an explorative celiotomy, to which consent was given. On reopening the cavity I proceeded to make a digital exploration of the sites of the ablated ovaries. On the right side I found a *multilocular cyst*, situated in a position normally occupied by the ovaries; in fact, adherent to the ligamentous tissue by which the ovary is suspended from the lumbar region. This was removed, and was proved to be ovarial tissue by microscopic examination.

Here, then, was a clear instance of a portion of *ovarial tissue*, but not necessarily a portion of the *ovary*, remaining behind after the first operation and persisting in its function.

I could find nothing of a cystic nature at the site of the left ovary. On this occasion I also removed the entire uterus. In April (1897), which would have been the time for the appearance of the next "heat" under normal conditions, there was no manifestation whatever of sexual appetite. The owner was careful to closely watch the animal, as we were all interested to see whether anything further were going to develop.

In October (1897) I was greatly astonished to have the same animal returned to me, acting in very much the same kind of way as Leeney's bitch—that is to say, she mated indiscriminately with anything that came along, but without exhibiting any sanguineous vaginal discharge. Such an extraordinary case was of rare occurrence, and the owner's interest being in nowise less than mine, I again opened the abdominal cavity, wondering whether there was not something after all in the British farriers' whim. But no; the root of the trouble was again the presence of a cyst, this time at the site of the extirpated left ovary. I removed it. From that period until the present the animal has behaved like a true neuter.

Case No. 2 of the fox-terrier bitches I have already referred to in the columns of the JOURNAL.[4] This animal was deprived of its ovaries in the fall of 1898, when some eight months of age. It estruated regularly thereafter until the spring of 1900, when heat occurred twice within three months—*i. e.*, there was then an irregular manifestation.

During the summer I operated a second time, and found a cyst present at the site of the left ovary. Not only had this animal mated with members of the opposite sex, but during the late spring, just prior to the second operation, it had actually fostered a kitten, yielding a copious supply of milk for the same. Beyond the fact that in this case the adopted offspring belonged to another species, there is nothing very remarkable about this. The presence of a cyst was proof of the persistence of ovarial tissue, and this continuing to function, the animal was still in the eye of the law of nature a female. The development of lactation without pregnancy is of quite common occurrence in bitches about the period parturition would be due. This phenomenon must be regarded as a mistaken anticipation of maternity on the part of nature.

Case No. 3 of the fox-terriers was deprived of her ovaries and also the uterus because it contained fœtuses. This animal was submitted to operation primarily because it had escaped from restraint during the previous (its first) estruation and had copulated with a dog much larger than itself. Being a highly regarded pet in the family that owned it, it was deemed advisable to do an early hysterectomy and remove the fœtuses, in order to guard against a possible mishap at full term. The owner, having decided objection to the unpleasantness of "heat" manifestation on the part of the animal, requested me when removing the fœtuses to place the animal beyond any possibility of again functioning.

Here was an animal in exactly the condition desired by the practitioner, Leeney, and the farriers. At the time of operating it had carried the fœtuses about fifteen days. Yet in spite of these conditions "heat" appeared at the next period and reappeared at irregular intervals (generally about three months) until I removed the source of the trouble, which was the same as in the other cases —persistence of ovarial tissue.

Cases Nos. 1 and 3 would seem to throw some light upon the physiology of reproduction in the female dog. Apparently, sexual desire in this animal is a reflex process dependent solely upon interstitial swelling in the ovaries. It is conceivable that the manner in which this takes place is through excitation of the terminal filaments of the ovarian nerves by pressure exerted by the distended ripening Graafian follicles. There would thus be carried to the higher nerve-centres the stimulus necessary to arouse the impulse to mate.

This hypothesis agrees in the main with that advanced by Pflüger[5] in 1865 to explain the mechanism of the uterine discharges. He believed that the latter depended upon reflex impulses resulting from growth of the ovarian cells and consequent swelling, and thereby stimulation of the ovarian nerve-fibres and through them the spinal cord. A reflex dilatation of the vessels and tumefaction in the genital organs was in turn produced.

It is an hypothesis which affords a reasonable explanation for the phenomenon of increase in frequency of or continuous estruation.

Granted the remaining behind of a small portion of ovarial tissue through imperfect operation, a local inflammatory reaction would be started which would lead to the formation of more or less cicatricial tissue on the free surface of the ovary (the ovary being attached by one face to the sac in which it lies). This cicatricial tissue would form a barrier to the rupture and escape of the ripening Graafian follicles at the next regular period of ovular development. The portion of tissue would still derive sufficient nutrition for functional purposes from its attached surface. Such follicles would be imprisoned, so to speak, and would coalesce. There would result therefrom a typical glandular retention cyst formation. Such cysts usually increase in size according to the rate of reproduction of the contained matter until finally they react on the source of their development by inducing a pressure atrophy of the secreting elements.

In the case of this persistent portion of ovary we may assume that it would comport itself exactly as does the tissue of the undisturbed ovary—that is to say, at intervals of regular periodicity there would occur development of ovules and formation of Graafian follicles. There is no reason to suppose that ovulation would continue without the periods of quiescence proper to the normal organ. But regular development would continue only for a limited time until the remaining ovarial tissue was "used up," so to speak, or was destroyed by pressure of accumulated unruptured follicles. With the ovarial tissue the nerve-endings would probably also eventually atrophy, and finally there would remain nothing but an inert mass consisting of a fibrous sac with cystic contents. And there being no longer impressionable nerve-endings, no stimulus could be received to awaken desire for sexual intercourse.

But what explanation can be offered for the period of quiescence exhibited in Case No. 1, where the portion of tissue belonging to the left ovary showed no signs of functioning from before the time I undertook the first operation (June, 1896) until I opened the cavity the second time (October, 1897)? I do not regard this as a matter of any wonder, for it is a fact that both ovaries do not necessarily function together at each estruation. This fact I have ascertained whilst examining the reproductive organs in the bodies of eighty-seven bitches destroyed at the city pound or which have died in my practice. Of this number sixteen were rutting at the time of their exit from the world, and in these sixteen I found two in which but one of the two ovaries present showed the presence of Graafian follicles, the other one being apparently quiescent. So that in the animal in question the portion of the left ovary remaining was probably in a state of rest during the period named.

Other instances have come to my notice of bitches functioning but once in twelve months.

Ovulation may also occur more frequently than usual when impregnation is prevented. I have in my possession a Great Dane bitch, three years of age, which has never succeeded in becoming impregnated, though she has been mated at each period. On one occasion of irregular estruation the interval between her periods was only nine weeks.

In my description of Case No. 1, I have remarked that the persistence of ovarial tissue after removal of both ovaries does not necessarily imply that portions of one or both of the latter have been left behind through imperfect operation. We must keep in mind the possibility of there being accessory ovaries (ovaria succenturiata). If it can be proved that such may and do sometimes exist, we do not need to seek further for an explanation of recurrent estruation after removal of the ovaries, for, as already remarked, the animal would still be possessed of sex, though incapable of bearing young, so long as ovarial tissue remained in the organism.

This matter of occurrence of accessory ovaries in the human female has been extensively investigated and discussed by both Thumin[6] and Engström.[7] Both these investigators have found them, and they cite numerous other observers who have recorded their occurrence. As a result of these observations an array of facts has been marshalled, the principal of which I will present to the reader: Accessory ovaries (in the human female) may have their origin either during fœtal or post-fœtal life, but they arise more commonly in post-fœtal life as the result of pathological changes in the ovary rather than during the embryonal existence. Their origin is most commonly traceable to inflammatory conditions in the ovary itself or to peritonitis, whereby bands of fibrous tissue traverse the gland and isolate or cut off portions of ovarial tissue from the main body. In ovaries which have functioned much deep depressions are sometimes found on the surface. These represent cicatricial tissue, the formation of which has followed the bursting of a Graafian follicle. Two cases are instanced where a sort of bridge of fibrous tissue divided the organ into two portions. They may also have their origin through torsion and consequent strangulation of the ligamentum ovarii and ligamentum latum, and through stretching of the ligamentum ovarii as a result of enlargement of the uterus.

Accessory ovaries developed in this manner are usually found to be attached to the ovary proper by ligamentous union, but sometimes they become completely detached and fall off into the peritoneal cavity, where they either become absorbed or attached to some other organ by adhesive inflammation and thus secure nutrition. In one instance one of these bodies was found attached to the large omentum. They usually occur singly, but may exist in multiples, six having been found in one case.

With regard to the frequency of their occurrence twenty-three cases were found in one lot of five hundred bodies examined and eighteen in another lot of five hundred, or an average of about 4 per cent.

It was with the object of ascertaining whether or not accessory ovaries might occur in the female dog that I undertook the examination of the reproductive organs of the eighty-seven animals referred to above. But in not a single instance could I find true segregation of ovarial tissue. The nearest approach to such a condition was visible in the ovaries of bitches advanced in years, the free surfaces of which exhibited mammilliform protuberances, the intervening sulci representing cicatrices which had resulted from the development and bursting of Graafian follicles.

Hence, I am led to the belief that if accessory ovaries do occur in the canine female it is with such extreme rarity that we are justified in dismissing them from consideration as being responsible for the phenomenon under discussion, and that this rarity may be attributed to the relative immunity of the organs from inflammatory disturbances, which is the most common cause of segregation of ovarial tissue in the human female.

Having thus, to my own satisfaction, disposed of the "third" ovary hypothesis, there remains to be considered but one other possible factor.

Is the central nervous system of the canine female capable of generating sexual impulse independently of the presence of the essential reproductive glands—the ovaries?

Let us glance at the conditions which prevail in the human female. Dr. J. Whitridge Williams, of the Johns Hopkins University, informs me that when oöphorectomy is performed on young girls (before puberty) the sexual appetite never occurs, whereas when it is performed on mature women the effect varies. In many cases the operation is followed by a gradual atrophy of the genital tract with complete disappearance of all sexual desire; whilst in others the operation appears to have no effect upon this function, and in a small number of cases seems to stimulate the desire.

Clearly, then, in women at least, sexual appetite is a function which may occur independently of any reflex stimulation resulting from ovulation, and if it may occur in the human female may it not also occur in the canine?

It must be remembered that amongst animals in a wild state ovulation is a periodic phenomenon occurring at certain seasons, and only at these seasons is there any sexual activity. Darwin has pointed out that domestication with its regular food supply and comforts has tended to increase productiveness in many domesticated animals and woman, and that the relation to seasons no longer exists. In other words, there has been an evolution of function until we find the highest type of female animal life—woman—a being possessed of remarkable reproductive capabilities and in whom sexual appetite may arise and be gratified independently of ovulation. Whether this latter function is to be considered an advance in evolutionary progress may be questioned. It might be equally as well regarded as a perversion of a natural instinct. As far as I am aware there is neither experimental or clinical evidence to support the view that similar "advanced" conditions may exist in the animal in question. Indeed, the evidence is all the other way. The mere observations of "heat" after oöphorectomy, which have been recorded at different times, are utterly valueless as evidence, for the simple reason that they have not been backed up by investigation, nor even by particulars as to the exact condition present.

Is the canine female ever seen to permit the approach of the male at any other time than during "heat," except under certain conditions which are manifestly abnormal? Are there any cases recorded of sexual desire being excited even by irritative processes localized in the vicinity of the erectile organs, such as medical literature tells us sometimes occurs in woman? I have repeatedly tried to awaken sexual desire in canine females by manipulating the clitoris, but absolutely without success. The nearest approach to such a condition that I have seen was in a little black-and-tan terrier, which would quite frequently indulge in spells of licking the vagina and would accompany this with slight copulatory motions, such as the male exhibits, until she appeared satisfied. But that this could not have been any real sexual appetite was evident from the fact that she utterly refused all advances on the part of the opposite sex excepting at her regular periods.

On the other hand, I have offered evidence to show that the persistence of minute portions of ovary after three instances of oöphorectomy or oöphoro-hysterectomy, where the operation was performed with ordinary care and supposedly to completion, has been responsible for exhibition of the sexual appetite. These three cases, and the one of the cat which I have not been able to investigate, represent all the instances which I have seen in my practice which, being confined to the canine species, is quite extensive, and I am of the opinion that they are typical of the experiences of other practitioners.

How may the experience of Cary be explained? Here was an instance of attractiveness on the part of a female which commenced almost immediately after operation, continued for about one year, and then ceased altogether, but with this difference—that the female herself entirely rejected the amatory attentions of the opposite sex. I think this question may be answered by referring to an authority on human gynecology. Glävecke[8] states that the vagina in the human female undergoes certain changes after castration. First, there occurs a marked hyperæmic injection of the mucosa, which soon becomes soft and swollen. Second, the normal secretion is appreciably increased, and at times an appearance is produced similar to pregnancy. This condition lasts for a few months, when it is succeeded by shrinkage and atrophy.

I have no doubt whatever that herein lies the explanation of Cary's case, as well as a good many of the other cases which have been rather vaguely reported as showing "signs of heat." There was no true estruation, but merely an extraordinary temporary sexual attractiveness of the female to the male, without, however, the former being a willing party thereto. The attractiveness is to be explained on the supposition of increased mucosal secretion, accompanied by the emission of a changed odor similar to that which invites the males at the estrual period.

I do not believe at all in the tradition of Leeney and the British farriers. Traditions are generally founded on some good grounds, but there are notable exceptions to the rule. What, for instance, can be said of the tradition which I have been solemnly informed by an ignorant farmer exists amongst some of the lower rural classes of the South, to the effect that fistulous withers in the horse may be cured by the simple operation known as "burying the sod." The technique of this very technical operation consists in leading the animal into a field. The owner then waits and watches and waits till that horse lies down and rolls. And wheresoever the diseased withers have touched the ground there he must dig up a sod and turn it over. If and when the grass grows through the other way the horse is cured. If this should fail to work, you fill the poor brute's ear with human excrement, and tie a string tightly round to keep it in! So much for myths. It is more than likely that the farriers aimed at excising simply the ovaries in the non-pregnant animal and failed to remove them in their entirety, whilst when they operated on pregnant animals they naturally extirpated the uterine cornua and part of the broad ligaments as well, and in removing the broad ligaments there was more likelihood of including the whole of each ovary.

If it be admitted that true estruation occurs only as a result of failure to remove the organs in their entirety, what can be the reason for such failure? Those conversant with the anatomy of the parts will remember that the ovary lies in a sort of sac—a duplicature of peritoneum, and is attached to the same by one of its faces. This enveloping tissue is often the seat of considerable fat, which may completely hide the organ within, so that its recognition *in situ* becomes a matter of impossiblity. If, in the performance of the operation, ligatures or scissors be employed, it is a very easy matter to leave behind a minute portion of the gland we are endeavoring to remove yet cannot see. I have always favored the employment of ligatures or emasculating devices in order to guard against any possible mishap from hemorrhage, but I am bound to admit that there is less chance of making an imperfect operation when the organ is torn from its renal and sublumbar attachments.

But even supposing that in making use of ligatures or cutting or crushing instruments a portion of ovary is left behind, such fact should always be discovered, for good surgery demands that a careful though necessarily rapid examination of the extirpated portions be made before the abdominal cavity is closed.

In view of the foregoing arguments I believe we may safely arrive at the following conclusions:

Complete oöphorectomy is an absolute preventative of subsequent estruation.

All instances of recurrence of true estruation after oöphorectomy are due to persistence of ovarial tissue, owing to incomplete operation. Such estruation appears first at the next regular period and recurs at each successive period, or may become continuous and finally cease altogether, the difference depending upon the amount of ovarial tissue left behind, or the development of a retention cyst in the same.

After complete oöphorectomy a false "heat" may sometimes be witnessed, the same depending upon increased and changed mucosal secretions, which are sufficient to attract the male but without concurrence on the part of the female. Such false "heat" can last only for a limited period.

REFERENDA.

1. Veterinary Journal, 1890, p. 11.
2. Bericht über das Veterinärwesen im Königreich Sachsen, 1868, p. 31.
3. JOURNAL OF COMPARATIVE MEDICINE AND VETERINARY ARCHIVES, 1900, p. 414.
4. Ibid., 1900, p. 693.
5. Untersuch. aus dem physiol. Labarat. zu Bonn, 1865.
6. Arch. für Gyn., 1898, p. 342.
7. Mittheil. der Gyn. Klinik des Prof. Engström, 1897, p. 55.
8. Arch. für Gyn., vol. xxv.

